# Concerning Amalgam Dental Plugs

**Published:** 1873-09

**Authors:** W. Henry King

**Affiliations:** Mount Sterling, Ill.


ARTICLE III.—*Concerning Amalgam Dental Plugs.* By W. HENRY KING, M.D., Mount Sterling, Ill.

Having just read, in the July number of the CHICAGO MEDICAL JOURNAL, an article on "Poisoning from corrosive sublimate generated in the mouth from amalgam plugs in the teeth," by J. Payne, D.D.S., of Dwight, Ill., I feel so "strangely queer," I scarcely know what *manner* of reply to attempt—and I would make none, were Dr. Payne's the first hollow notes set to the same inharmonious tune. I hardly know how or where "to take him," or what to do with him after the taking. Emotions crowd so thick and fast, I know not what to do. "You'd scarce expect one of my age"—no, that won't do. Well, "Fools rush in where angels dare not tread"—no, nor that either.

> "O, were you ne'er a schoolboy,
> And did you never feel
> That swelling of the heart
> You ne'er shall feel again?"

And now I fancy "commencement day," "pigeon-tailed" coats, ribbons—and parchment paper done up in tin canisters; and backward yet to primer days, and the rudiments of chemistry and other *trifles*, memory wings her course. But, hold! My pegasus is about getting entirely away with me. I was about to remark, I am a very modest man, and do not like, as some people do, to parade myself unnecessarily before the public. I am an humble individual, and no "eminent gentleman of the medical profession" ever condescended to pay any particular attention to me, and so my little effort must "go on its own hook." There is, however, nobody in this locality much alarmed about "amalgam plugs," but lest there be an epidemic of fright in the vicinity of Dwight, we propose to take down that scare-crow, dissect it, and ascertain, if possible, whether the altitude it reached in its dizzy flight was due to its light frame-work, or to excessive muscular development at the expense of intellectual training, and as we pass along, *laugh* where we must, and be earnest where there's room—always adapting, in our hunt, our ammunition to the target raised.

And, first of all, years of *experience* warn me not to advocate the use of amalgam plugs, *only in exceptional cases.* They are to be condemned for general use, but on altogether different grounds from the ensuing chemical combinations claimed by Dr. Payne.

Dr. Payne says, "every medical man, of any considerable practice, has undoubtedly had numerous cases of it," *i. e.*, "poisoning from corrosive sublimate generated in the mouth from amalgam plugs in the teeth, *but never knew what it was.*" No doubt! No doubt wisdom and knowledge are on the increase, and revolve around Dwight as the great centre or fountain of all scientific light! No doubt the general ignorance of medical gentlemen, during the past fifty years of "amalgam plugs," has been so intense that they left this great discovery of "plug" poisoning to Dr. Payne, while they were busy treating patients suffering from "so numerous and varied symptoms" for all the troubles that gentleman has catalogued!

Again, he says: "The patient gradually wastes away as if going into a decline," [how 's that! has Barnum got one?] "*and no medicine will afford any relief.*" No wonder! Sic. Send for McGinnis. Now it is a pity Dr. Payne "had not time to delineate *the manner* in which corrosive sublimate is formed in the mouth," [and to give us the symbol and atomic weight of the new *element*, "any saline substance, such as food,"] "further than to say that the quicksilver in the plugs is driven off by the heat of the mouth in minute particles, and combining with the chlorine in the fluids of the mouth, *or any saline substance, such as our food*, passes into the stomach, and produces slow poisoning."

*Take time*, Dr. Payne, and describe this process scientifically, and rival Jenner by your fame!

Now what are the facts in the case? Let us briefly state them.

1. The symptoms Dr. Payne describes, in the main, do not appertain to the mercurial diathesis.

2. If the state of health described were induced by mercury, every respectable practitioner knows remedies that will relieve it, if Dr. Payne don't.

3. "Amalgam plugs in the teeth" do not disturb or derange the health of *any one* in the slightest degree on account of the mercury they contain, directly or indirectly, and no one dare attempt to sustain such a proposition *but a homœopathist*, and even he would blush at the result.

Let us use figures—not bombast—and approximate the truth. Yesterday, I removed four fillings from grinding surfaces after they were worn in different mouths for seven years. This number of fillings (four) would be a very large average for one mouth to contain. The aggregate weight at the time of insertion was thirty-six grains, viz.: silver and tin, twenty-two grains; mercury, fourteen grains. The weight seven years after: silver and tin, twenty-one grains; mercury, twelve. That is, four plugs in seven years, or 2,556 days, lose two grains of mercury, or one grain in 1,278$=\frac{1}{1278}$ grain of mercury per day. Now, if, after the plug has once become hard, mercury escapes into the mouth, it must do so through the agency of heat, and as the mouth has a *constant* temperature of 98.4°, the escape of mercury must be *constant*. How, then, since mercury, *i. e.*, bichloride of mercury, is *not* cumulative, will Dr. Payne explain these *sudden* "poisonings and prostrations"?

Mercury, as mercury, is inert. Chlorine is found in the saliva, only in combination with potassium and sodium, and only acts on mercury when in excess. Now, does the chlorine give up the powerful bases, potassium and sodium, for mercury? We deny it. But suppose it did, how much bichloride of mercury would $\frac{1}{1278}$ of a grain of mercury form daily? Let us see. Atomic weight of Hg.=100 of Cl.=35.5. Then 271 : 200 : : 1 : amount of Hg. in one grain Hg. Cl.; or one grain Hg. Cl.=.73 parts mercury, plus .27 chlorine; or one grain of mercury will make 1.37 grains corrosive sublimate, and .1278 grain of mercury would make .932 of a grain of corrosive sublimate in the mouth daily. Now, allow $\frac{1}{5}$ of a grain of corrosive sublimate as the ordinary dose, it would only take 186 days to get a dose in the manner described by Dr. Payne. Now, if all this will not answer this much mooted question, look at it another way. If you expel *all* the mercury from a plug by a slow low temperature, the remaining metals will be but a fine powder; so as long as a filling remains solid *it contains mercury*. I saw an amalgam plug to-day that still *fills* up the cavity in which it was placed *thirty years ago*, and it remains dense and hard. This filling, according to the proportional amount of mercury required at the time of insertion, could not have contained more than four grains of mercury and still holds some, and just such fillings have been worn for sixty years, without harm, and will be worn again, long after friend Payne has gone "where the woodbine twineth."

In conclusion, allow me to recommend the State Medical Society to send a committee to Dwight in search of candidates for Jacksonville, and to petition the next General Assembly for a law to hang *every man* who knows of 12,000 people daily destroying more lives than all the cholera, etc., in the land, *and will not take "time to delineate the manner"* in which the poison is formed, though he knows it to a certainty. "Let us have peace." What next?

